# Correction: Tracking ebolavirus genomic drift with a resequencing microarray

**DOI:** 10.1371/journal.pone.0285149

**Published:** 2023-04-26

**Authors:** Irina Tiper, Moussa Kourout, Bryan Lanning, Carolyn Fisher, Krishnamurthy Konduru, Anjan Purkayastha, Gerardo Kaplan, Robert Duncan

## Notice of Republication

This article was republished on April 17, 2023, to add Bryan Lanning as the third author. The Funding Statement, About the Authors, and citation details were updated accordingly. Please download this article again to view the correct version.

## Supporting information

S1 File Originally published, uncorrected article. (PDF)

S2 File Republished, corrected article. (PDF)
