# Supplementary material for: Tracking ebolavirus genomic drift with a resequencing microarray
Source: PLoS One. 2022 Feb 10;17(2):e0263732. doi: 10.1371/journal.pone.0263732 (PMC8830711; doi:10.1371/journal.pone.0263732)
Supplement: S2 Table — Oligonucleotide primer sequences are in a table in a Microsoft Word document. (DOC) [file pone.0263732.s002.doc]

| Tile-Primer ID | Primer Pool | Primer Sequence |
| --- | --- | --- |
| >FILO_EBOV_NP-1 | MixA | AGGGTGATCCAACAACCTTAAT |
| >FILO_EBOV_NP-2 | MixA | CTGTCCGCACTCTCTTGAAA |
| >FILO_EBOV_NP-3 | MixA | AAGAGAGTGCGGACAGTTTC |
| >FILO_EBOV_NP-4 | MixA | GTCCTTGCTCTGCATGTACT |
| >FILO_EBOV_NP-5 | MixA | GCAGAGCAAGGACTGATACAA |
| >FILO_EBOV_NP-6 | MixA | AAAGGAGCATACTCTCCATGC |
| >FILO_EBOV_NP-7 | MixA | CTCCTTTCGCCCGACTTT |
| >FILO_EBOV_NP-8 | MixA | GGAATGGTCGTATCCTGTGAG |
| >FILO_EBOV_NP-9 | MixA | GATACGACCATTCCCGATGTG |
| >FILO_EBOV_NP-10 | MixA | AAGTTCCGTCCCTGTCCT |
| >FILO_EBOV_NP-11 | MixA | GGAGATGTATCGCCACATTCTAA |
| >FILO_EBOV_NP-12 | MixA | CGCCTATTTGCATTAAGACTCAAA |
| >FILO_EBOV_vp40-1 | MixA | CCAAGCAATAATGACTTCACTCC |
| >FILO_EBOV_vp40-2 | MixA | CAATGGTGTAAAGCACCTTTAGTAT |
| >FILO_EBOV_GP-1 | MixA | TATCAGGCTACCGGTTTTGG |
| >FILO_EBOV_GP-2 | MixA | GCTGTGCTGTCGTTGTCTGT |
| >FILO_EBOV_L-1 | MixA | GATTAAATCGAGGAAACTCTAGATCAAC |
| >FILO_EBOV_L-2 | MixA | AAGTAATCTCCGCTCTGGTAAAG |
| >FILO_EBOV_L-3 | MixA | CCACACTCCCATGTATGATTGA |
| >FILO_EBOV_L-4 | MixA | CGATTGGCCATGAAAGAATGAG |
| >FILO_EBOV_L-5 | MixA | ATGCAGTATTCGAGCCTAATGT |
| >FILO_EBOV_GP_VSV-1 | MixA | GTCTCTCCTAATTCCAGCCTC |
| >FILO_EBOV_GP_VSV-2 | MixA | GCCCATTCACCAGCTTCATA |
| >FILO_EBOV_L-6 | MixA | CAAAGCTACTCCCTCTAACTGTG |
| >TIM_Control-1 | MixA | ATGGCAGCTACCTCTCTCACTG |
| >TIM_Control-2 | MixA | CAAGCTATCACTCCAAGACC |
| >FILO_EBOV_GP-3 | MixB | ACAACCCTTGCCACAATCTC |
| >FILO_EBOV_GP-4 | MixB | GCACTCTCTTCTCCGGTATCT |
| >FILO_EBOV_GP-5 | MixB | GGTCAATTATGAAGCTGGTGAATG |
| >FILO_EBOV_GP-6 | MixB | AGCTGGAGCAGAAACTGTG |
| >FILO_EBOV_L-7 | MixB | ACAACATACCCAATACCCAGAC |
| >FILO_EBOV_L-8 | MixB | AGAGTTTCCTCGATTTAATCTACCC |
| >FILO_EBOV_L-9 | MixB | CATCCCGAATAAGCTGGACTATC |
| >FILO_EBOV_L-10 | MixB | CGGGTGCAACACTTAGTTAGA |
| >FILO_EBOV_L-11 | MixB | CACATCGCTCATTGCGAATAC |
| >FILO_EBOV_L-12 | MixB | TGTGGATCTCTGGTGGTTTG |
| >FILO_EBOV_L-13 | MixB | CTGCCATTGACTTTGTGCTAAA |
| >FILO_EBOV_L-14 | MixB | GGGAGTGTGGCTCCAATAAG |
| >FILO_EBOV_L-15 | MixB | TTACAAGTGCCTGTGGAATCT |
| >FILO_EBOV_L-16 | MixB | CCAGGGTTCTTCGCAATTAAAG |
| >FILO_EBOV_L-17 | MixB | CAAAGTCGGACTCAAACATATCAC |
| >FILO_EBOV_L-18 | MixB | AACCAACCTTTGCAAGTATCAG |
| >NAC_Control2-1 | MixB | AAATCATGGAGACGGAAGAAGA |
| >NAC_Control2-2 | MixB | AGCTTCCCATGTTGTCTCTA |
| >FILO_EBOV_vp40-3 | MixC | TTCGGCAAGGCAACCAAT |
| >FILO_EBOV_vp40-4 | MixC | GGAAGACTTGCAGGAGAATGAC |
| >FILO_EBOV_vp40-5 | MixC | TACCTCGGCTGAGAGAGTG |
| >FILO_EBOV_vp40-6 | MixC | CCTTGCCGAAATGGGTGATA |
| >FILO_EBOV_GP-7 | MixC | CCGACAGTGAGCGTAATCTTC |
| >FILO_EBOV_GP-8 | MixC | GCCCATTCACCAGCTTCATA |
| >FILO_EBOV_L-19 | MixC | CTTACTCTCCATATTAGGGTCTGATG |
| >FILO_EBOV_L-20 | MixC | CAACGGAGGGAATTGATTTCTTT |
| >FILO_EBOV_L-21 | MixC | CACAGTTAGAGGGAGTAGCTTTG |
| >FILO_EBOV_L-22 | MixC | ATTCCACAGGCACTTGTAACT |
| >FILO_EBOV_L-23 | MixC | CATGTAAGAGTACAGCCAGCAA |
| >FILO_EBOV_L-24 | MixC | CTGTGGTATCAATGACGGATCTC |
| >FILO_EBOV_L-25 | MixC | CTCCGAATGATTGAGATGGATGA |
| >FILO_EBOV_L-26 | MixC | GCTAATAATGTGCGTGTTCCTTC |
| >FILO_EBOV_L-27 | MixC | ACAACTCAGCAAGCCAAATAAC |
| >FILO_EBOV_L-28 | MixC | GTGATATGTTTGAGTCCGACTTTG |
| >TIM_Control-3 | MixC | TGAGGGTCTTGGAGTGATAG |
| >TIM_Control-4 | MixC | GGTCGCTAACCTCCCTTTTATT |
| >FILO_EBOV_NP-13 | MixD | CCAAGGGTGGACAACAGAA |
| >FILO_EBOV_NP-14 | MixD | GTGGCGATACATCTCCTCAAA |
| >FILO_EBOV_GP-9 | MixD | CTGGCAACAACAACACTCATC |
| >FILO_EBOV_GP-10 | MixD | CGGTATCCATTGTCTCCATCC |
| >FILO_EBOV_L-29 | MixD | CCACAACAACTTTGTGAGCTATT |
| >FILO_EBOV_L-30 | MixD | CTACCTACATTCAACTCTTTCTCTTTC |
| >FILO_EBOV_L-31 | MixD | GCAGTTGCACTGTTCGATATT |
| >FILO_EBOV_L-32 | MixD | CAATGAGCGATGTGGTAGATTATG |
| >FILO_EBOV_L-33 | MixD | CGGCAATTGAGATCCGTCATT |
| >FILO_EBOV_L-34 | MixD | GTAGGATTTGTTATGTCTGTTATTTGGC |
| >NAC_Control-3 | MixD | GGAAGACTGGGTCTTGTGTA |
| >NAC_Control-4 | MixD | TGTACAATCAGACAAGCACACG |

Primer Pair Substitutions for TAFV/BDBV Pools

| Tile-Primer ID | Substituted TAFV Primer Sequence | EBOV Primer removed | Pool |
| --- | --- | --- | --- |
| TAFV_VP40_F1 | TTCAGTTGCAAGGAGCTA | >FILO_EBOV_vp40-5 | MixC |
| TAFV_VP40_R1 | GCCAAAATGAGTGATGGTAT | >FILO_EBOV_vp40-6 | MixC |
| TAFV_GP_F7 | GCAACCAGTGGTGCTATCT | >FILO_EBOV_GP-7 | MixC |
| TAFV_GP_R8 | CCATTCTCCAGCTTCGCA | >FILO_EBOV_GP-8 | MixC |
| TAFV_GP_F5 | CCAAAGGTGGTAAATTGCGA | >FILO_EBOV_GP-5 | MixB |
| TAFV_GP_R6 | TCTGCGGTTATCAGAGTAG | >FILO_EBOV_GP-6 | MixB |
| TAFV_GP_F1 | TACGTGGTTGATAATTTTGG | >FILO_EBOV_GP-1 | MixA |
| TAFV_GP_R2 | GTGTCGTCGATTCTGTG | >FILO_EBOV_GP-2 | MixA |
| TAFV_GP_F9 | CCAGAACAGCACACTGCC | >FILO_EBOV_GP-9 | MixD |
| TAFV_GP_R10 | AGGAACCCATTGTTTCCATCC | >FILO_EBOV_GP-10 | MixD |
| TAFV_GP_F3 | CAACAACCACACCCTCTCCA | >FILO_EBOV_GP-3 | MixB |
| TAFV_GP_R4 | CCAGGTCCACTGAGTTCGTC | >FILO_EBOV_GP-4 | MixB |
| TAVF_L_F31 | GCTGTTGCACTTTTTGATCTAC | >FILO_EBOV_L-31 | MixD |
| TAFV_L_R18 | GGTACCATTGTATAACTATGTGGAG | >FILO_EBOV_L-18 | MixB |

Primer Pair Additions for rVSV-EBOVgp-GFP Pools

| Tile-Primer ID | Substituted rVSV-EBOVgp GFP Primer Sequence | EBOV Primer removed | Pool |
| --- | --- | --- | --- |
| VSVGP_001_F1 | GTCTCTCCTAATTCCAGCCTC | none | MixD |
| VSVGP_002_R1 | GCCCATTCACCAGCTTCATA | none | MixD |
