# Supplementary figures and images for: Tracking ebolavirus genomic drift with a resequencing microarray
Source: PLoS One. 2022 Feb 10;17(2):e0263732. doi: 10.1371/journal.pone.0263732 (PMC8830711; doi:10.1371/journal.pone.0263732)

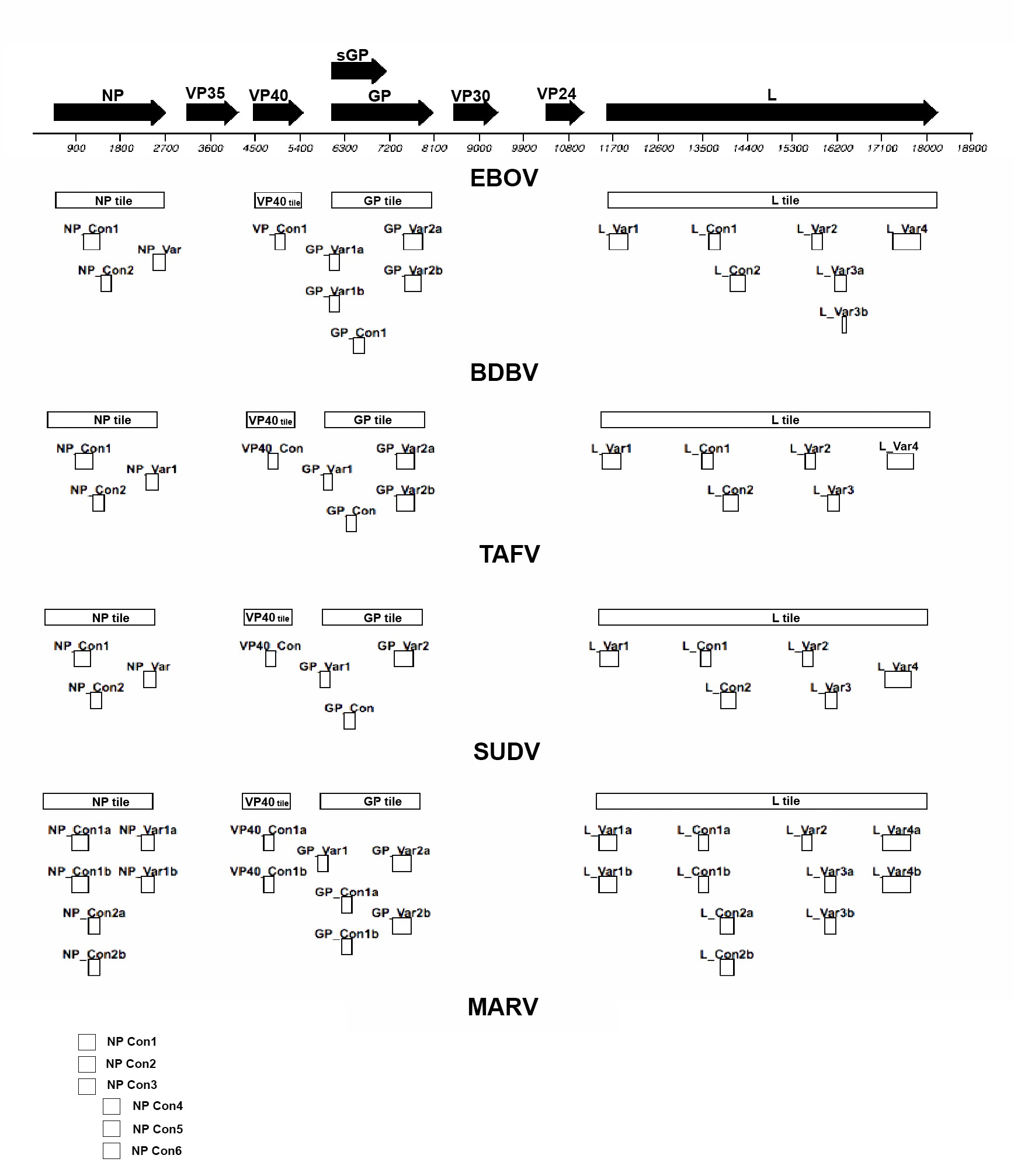

Supplement: S1 Fig — The genome and the eight coding sequences of Ebolaviruses are shown at the top. Beneath and aligned with the sequence content, are the tiles for each Ebolavirus. Note that greater variability among Sudan ebolavirus genomes required additional tiles, each with slightly different sequence composition. The microarray also has six tiles in the NP region of Marburg virus that are intended to detect a related Filovirus that may be the causative agent of disease symptoms in Ebola-negative patients. (TIF) [file pone.0263732.s003.tif]

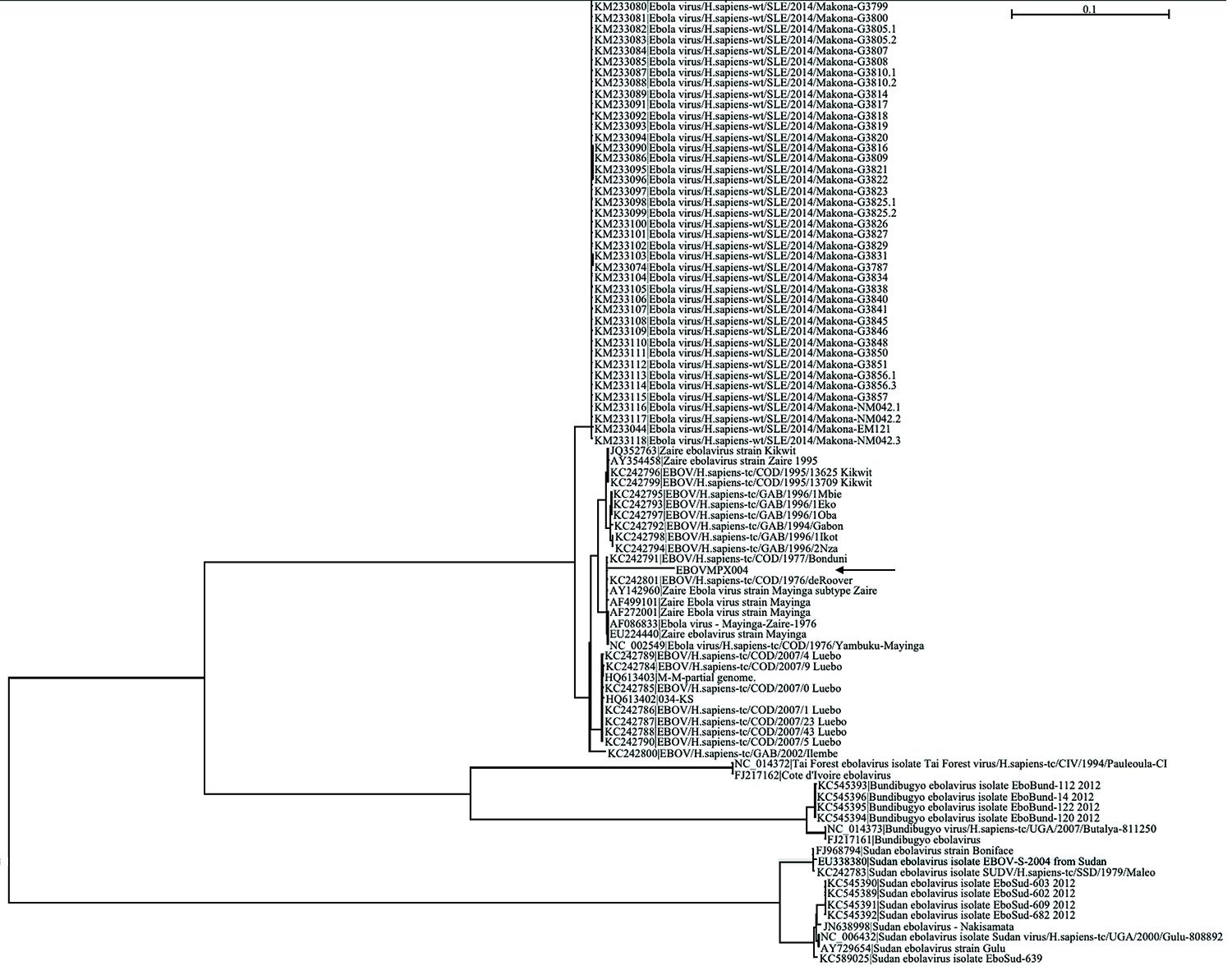

Supplement: S2 Fig — An RNA sample of EBOV Mayinga isolate (BEI Resources, NR-31806), sequenced with the Ebolavirus-RMA (chip #EBOVMPX004). The sequence output was processed with the ebola_i2o pipeline. The phylogenetic tree output of the pipeline is visualized with SeaView Version 4.7 [33]. Each file in the tree carries the name assigned to that file in the database, FilovirDB. According to this tree, the genomes in FilovirDB most closely related to the Ebolavirus-RMA output sequence are KC242791, KC24801, AF499101 and AY142960. AY142960 is the database sequence entry for the BEI Resources NR-31806 sample. An arrow indicates the location on the tree of the RMA output sequence. This figure truncates the list of Ebola virus Makona sequences because the large number of sequences in the database from the 2014–2016 West African outbreak, all very similar to each other, are not necessary to display in this figure to demonstrate their phylogenetic relationship. Horizontal bar indicates the branch length equal to 0.1 phylogenetic units (approximately 10% difference). (TIF) [file pone.0263732.s004.tif]
