# Supplementary material for: Tracking ebolavirus genomic drift with a resequencing microarray
Source: PLoS One. 2022 Feb 10;17(2):e0263732. doi: 10.1371/journal.pone.0263732 (PMC8830711; doi:10.1371/journal.pone.0263732)
Supplement: S1 File — (DOCX) [file pone.0263732.s005.docx]

**Author Contributions**

| **Author** | **Role** | **Affiliation** | **Definition** |
| --- | --- | --- | --- |
| Irina Tiper  [Irina.tiper@fda.hhs.gov](mailto:Irina.tiper@fda.hhs.gov)  10903 New Hampshire Ave | Formal Analysis | Division of Emerging and Transfusion-Transmitted Diseases, Office of Blood Research and Review, Center for Biologics Evaluation and Research, US Food and Drug Administration, Silver Spring, MD, USA, | Application of statistical, mathematical, computational, or other formal techniques to analyze or synthesize study data. |
|  | Investigation | FDA/CBER | Conducting a research and investigation process, specifically performing the experiments, or data/evidence collection. |
|  | Writing – Review & Editing | FDA/CBER | Preparation, creation and/or presentation of the published work by those from the original research group, specifically critical review, commentary or revision – including pre- or post-publication stages. |
| Moussa Kourout  moussa.kourout@fda.hhs.gov | Conceptualization | FDA/CBER | Ideas; formulation or evolution of overarching research goals and aims |
|  | Formal Analysis | FDA/CBER | See above |
|  | Investigation | FDA/CBER | See above |
| Carolyn Fisher  Carolyn.fisher@fda.hhs.gov | Investigation | FDA/CBER | See above |
|  | Writing – Review & Editing | FDA/CBER | See above |
| Krishnamurthy Konduru  Krishnamurthy.Konduru@  fda.hhs.gov | Investigation | FDA/CBER | See above |
|  | Writing – Review & Editing | FDA/CBER | See above |
| Anjan Purkayastha  anjan.purkayastha@gmail.com | Software | OpenBox Bio, Vienna, VA, USA | Programming, software development; designing computer programs; implementation of the computer code and supporting algorithms; testing of existing code components. |
|  | Conceptualization | OpenBox Bio, Vienna, VA, USA | See above |
| Gerardo Kaplan  Gerardo.kaplan@fda.hhs.gov | Supervision | FDA/CBER | Oversight and leadership responsibility for the research activity planning and execution, including mentorship external to the core team |
| Robert Duncan | Conceptualization | FDA/CBER | See above |
|  | Formal Analysis | FDA/CBER | See above |
|  | Funding Acquisition | FDA/CBER | Acquisition of the financial support for the project leading to this publication. |
|  | Project Administration | FDA/CBER | Management and coordination responsibility for the research activity planning and execution. |
|  | Writing – Original Draft Preparation | FDA/CBER | Creation and/or presentation of the published work, specifically writing the initial draft (including substantive translation). |
|  | Writing – Review & Editing | FDA/CBER | See above |

**Funding Source:** Irina Tiper and Moussa Kourout were supported in part by an appointment to the Oak Ridge Institute for Science and Education (ORISE) Research Fellowship Program at the Center for Biologics Evaluation and Research administered by the ORISE through an interagency agreement between the U.S. Department of Energy and the U.S. Food and Drug Administration.

Funding for this research was partially provided by the FDA Medical Countermeasures Initiative (MCMi). The funders had no role in study design, data collection and analysis, decision to publish, or preparation of the manuscript.
