# Supplementary material for: Correction: Tracking ebolavirus genomic drift with a resequencing microarray
Source: PLoS One. 2023 Apr 26;18(4):e0285149. doi: 10.1371/journal.pone.0285149 (PMC10132641; doi:10.1371/journal.pone.0285149)
Supplement: S2 File — (PDF) [file pone.0285149.s002.pdf]

PLOS ONE

RESEARCH ARTICLE

Tracking ebolavirus genomic drift with a resequencing microarray

Irina Tiper[1☯¤], Moussa Kourout[1☯], Bryan Lanning[1☯], Carolyn Fisher[1], Krishnamurthy Konduru[1], Anjan Purkayastha[2], Gerardo Kaplan[1], Robert Duncan[1]*

1 Division of Emerging and Transfusion-Transmitted Diseases, Office of Blood Research and Review, Center for Biologics Evaluation and Research, US Food and Drug Administration, Silver Spring, MD, United States of America, **2** OpenBox Bio, Vienna, VA, United States of America

☯ These authors contributed equally to this work.
¤ Current address: Center for Biologics Evaluation and Research, Office of Tissues and Advanced Therapies, Division of Cell and Gene Therapies, US Food and Drug Administration, Silver Spring, MD, United States of America
* robert.duncan@fda.hhs.gov

Check for updates

OPEN ACCESS

Citation: Tiper I, Kourout M, Lanning B, Fisher C, Konduru K, Purkayastha A, et al. (2022) Tracking ebolavirus genomic drift with a resequencing microarray. PLoS ONE 17(2): e0263732. https://doi.org/10.1371/journal.pone.0263732

Editor: Jishnu Das, University of Pittsburgh, UNITED STATES

Received: July 2, 2021

Accepted: January 25, 2022

Published: February 10, 2022

Copyright: This is an open access article, free of all copyright, and may be freely reproduced, distributed, transmitted, modified, built upon, or otherwise used by anyone for any lawful purpose. The work is made available under the Creative Commons CC0 public domain dedication.

Data Availability Statement: The complete description of the microarray is available at ArrayExpress (https://www.ebi.ac.uk/arrayexpress/experiments/) with the accession number A-MTAB-670. The details of microarray results including the image file (CEL files), base-calling file (CHP) and the final called base sequence (txt files) of each microarray are available at ArrayExpress (https://www.ebi.ac.uk/arrayexpress/) under the accession number E-MTAB-10007 for the Ebola genome resequencing. The raw Illumina NGS reads and the assembled consensus

Abstract

Filoviruses are emerging pathogens that cause acute fever with high fatality rate and present a global public health threat. During the 2013–2016 Ebola virus outbreak, genome sequencing allowed the study of virus evolution, mutations affecting pathogenicity and infectivity, and tracing the viral spread. In 2018, early sequence identification of the Ebolavirus as EBOV in the Democratic Republic of the Congo supported the use of an Ebola virus vaccine. However, field-deployable sequencing methods are needed to enable a rapid public health response. Resequencing microarrays (RMA) are a targeted method to obtain genomic sequence on clinical specimens rapidly, and sensitively, overcoming the need for extensive bioinformatic analysis. This study presents the design and initial evaluation of an ebolavirus resequencing microarray (Ebolavirus-RMA) system for sequencing the major genomic regions of four Ebolaviruses that cause disease in humans. The design of the Ebolavirus-RMA system is described and evaluated by sequencing repository samples of three Ebolaviruses and two EBOV variants. The ability of the system to identify genetic drift in a replicating virus was achieved by sequencing the ebolavirus glycoprotein gene in a recombinant virus cultured under pressure from a neutralizing antibody. Comparison of the Ebolavirus-RMA results to the Genbank database sequence file with the accession number given for the source RNA and Ebolavirus-RMA results compared to Next Generation Sequence results of the same RNA samples showed up to 99% agreement.

Introduction

Filoviruses are emerging pathogens that cause acute fever with high fatality rate and present a public health threat that impacts far outside the immediate area of an outbreak. The average case fatality rate is around 50% and has been reported to be as high as 90% [1]. There are four Ebolaviruses which cause the most severe disease in humans: Ebola virus (EBOV), Bundibugyo

sequence are available at ArrayExpress (https://www.ebi.ac.uk/arrayexpress/) with the accession number E-MTAB-10102. The details of microarray results including the image file (CEL files), base-calling file (CHP) and the final called base sequence (txt files) of each microarray are available at ArrayExpress (https://www.ebi.ac.uk/arrayexpress/) under the accession number E-MTAB-10008 for the resequencing of the Ebola glycoprotein gene inserted into Vesicular Stomatitis Virus. The initial stock RNA was also subject to Illumina next generation sequencing for comparison to the Ebolavirus-RMA consensus sequence. The raw reads and the assembled consensus sequence are available at ArrayExpress (https://www.ebi.ac.uk/arrayexpress/) with the accession number E-MTAB-10157. The Round 3 RNA sample was also submitted to Illumina HiSeq NGS and the EBOV glycoprotein assembled for comparison with the RMA result. The raw reads and the assembled consensus sequence are also available at ArrayExpress under the accession number E-MTAB-10157.

Funding: Irina Tiper, Moussa Kourout and Bryan Lanning were supported in part by an appointment to the Oak Ridge Institute for Science and Education (ORISE) Research Fellowship Program at the Center for Biologics Evaluation and Research administered by the ORISE through an interagency agreement between the U.S. Department of Energy and the U.S. Food and Drug Administration. Funding for this research was partially provided by the FDA Medical Countermeasures Initiative (MCMi). Received by RD There is no grant number. https://www.fda.gov/emergency-preparedness-and-response/counterterrorism-and-emerging-threats/medical-countermeasures-initiative-mcmi. The funders had no role in study design, data collection and analysis, decision to publish, or preparation of the manuscript.

Competing interests: The authors have declared that no competing interests exist.

Abbreviations: RMA, Resequencing microarray; PCR, Polymerase Chain Reaction; EBOV, Ebola virus; BDBV, Bundibugyo virus; SUDV, Sudan virus; TAFV, Taï Forest virus.

virus (BDBV), Sudan virus (SUDV) and Taï Forest virus (TAFV) [2, 3]. EBOV has been responsible for the largest outbreaks, with the most widespread epidemics occurring recently in Central Africa. In West Africa, in late 2013 and early 2014, the level of concern for Ebola virus disease (EVD) increased because it spread quickly, reached densely-populated areas, and persisted for the longest duration, resulting in 28,639 infections and 11,316 deaths [4]. An epidemic in Central Africa presented additional serious concerns, because, like the 2013–2016 epidemic, it was affecting urban areas, as well as areas of active conflict. Beginning in August 2018 in the North Kivu region of the Democratic Republic of the Congo, the outbreak was also extended in duration compared to pre-2013 outbreaks and was declared over on 25 June 2020 with 3,470 total cases and 2,287 deaths [5]. Part of the success in controlling the epidemic was the availability of a vaccine based on recombinant, replication competent, vesicular stomatitis virus expressing a surface glycoprotein of Ebola virus now licensed by the US FDA called rVSV-ZEBOV-GP, first utilized in ring vaccination trials during the previous epidemic [6]. As of July 2020, 303,000 people had been vaccinated. Another outbreak in the Democratic Republic of the Congo, this time in the Equateur Province, shown by sequencing to be a unique variant, was brought under control by November 2020 [7]. The continuous threat of Ebola Virus Disease is manifest in a new outbreak in Guinea, beginning January 18, 2021 [8]. Virus sequences posted to a pre-print sequence site March 12, 2021 indicate the infections are a resurgence of the West African outbreak of 2013–2016 [9, 10].

The importance of the current study is based on the role of genome sequencing in each of these Ebola outbreaks, During the 2013–2016 ebolavirus outbreak, more viral genomes were sequenced than ever before, thus allowing the study of the evolution of the virus [11]. An evolution that was demonstrated to be continuous yet varied among Ebolavirus species [12]. While valuable information about the disease was gained from the sequences, limited amount of analyzed sequencing data was available in the early stages of the epidemic to aid in monitoring the outbreak. The sequencing information could have been used to establish the genetic and epidemiological factors that promoted the rapid and unchecked spread of the virus. Genetic changes that would affect diagnostics or therapeutics, were not available until the epidemic advanced into its later stages when more sequences were obtained, and continued monitoring is important to ensure no further mutations arise in ensuing outbreaks [13, 14]. Mutations affecting pathogenicity and infectivity, unique to the Makona EBOV isolate (from Guinea), were identified, although there were few such examples, and it remains controversial to what extent the mutations directly affect the phenotypes of the viruses in animal models [15].

Rapid identification of the Filovirus in the Democratic Republic of the Congo [16] as EBOV by Next Generation Sequencing (NGS) supported the use of a rVSV-ZEBOV-GP vaccine which was important in controlling that outbreak. The need for rapid, accessible sequence data is indicated by the identification of the variant in the most recent outbreak in Guinea as a resurgence of the earlier outbreak thought to have ended in 2016 [9, 10]. Together, these studies demonstrate the value of sequencing methods that can be located close to the outbreak to quickly generate real-time sequencing data without the need for a large bioinformatics infrastructure.

Resequencing microarray (RMA) chips are a targeted method to obtain genomic sequence on clinical specimens accurately and sensitively [17, 18]. The *in situ* synthesized GeneChip® RMAs (Affymetrix, Inc., Santa Clara, CA) are extremely high-density microarrays of oligonucleotide probes. Short fluorophore-labeled DNA fragments (20–200 bp) amplified from the sample, hybridize to these probes. After image capture of these hybridization events and immediate image analysis, base-pair resolution of the sample sequence is produced [19]. RMAs are a proven method for detection and diagnosis of viral disease in a highly multiplexed

format, enabling screening of 30 or more pathogens in parallel with rapid turnaround time [20, 21]. RMAs do not require the complex assembly software or internet connectivity as NGS does for sequence generation, thus enabling data analysis to be completed at a laboratory in the country of the outbreak, rather than relying on data collection at the site and data analysis in another country. The time between completion of wet laboratory protocols and generation of final sequence is as short as 5 hours, with interpretation of data accomplished by moderately trained laboratory personnel. Whole genome sequences of RNA and DNA viruses, including EBOV, have been reported using RMA systems [22, 23].

This study presents the design and initial evaluation of an ebolavirus resequencing microarray (Ebolavirus-RMA) system that demonstrates sequencing the major genomic regions of four Ebolaviruses known to cause disease in humans and reveals the ebolavirus glycoprotein coding sequence changes as a recombinant virus is passaged under pressure from a neutralizing antibody.

Materials and methods

Resequencing microarray design

A comprehensive survey of the available ebolavirus genomes was performed, and computational methods employed to identify the genomic regions with the highest variability and least variability across the known genomes of EBOV, BDBV, SUDV and TAFV. Along with a literature survey to determine genome regions of known importance, the results of the alignment analysis guided the design of sequence regions (tiles) to be manufactured on the microarray. The resequencing microarray designed has an assembly of tiles covering every base in the selected coding sequences of the four evaluated Ebolaviruses. For computational efficiency, seven short variable region tiles, and six short, conserved region tiles were designed for each Ebolavirus that overlap the coding sequence tiles with a total of 52 short tiles to cover the four Ebolaviruses. Some of the short tiles have multiple slightly different copies to ensure accurate sequence determination where higher variability among known isolates required. Fig 1 illustrates the position of the sequence of those tiles aligned with the EBOV genome sequence. The aligned position of all the microarray tiles is shown in S1 Fig. Six short tiles designed in the NP region ensured detection of the known variants of the Marburg Filoviruses (S1 Fig), though they were not tested experimentally. The array design was sent to Affymetrix, Inc. for manufacturing as previously described [24].

Each tile detector is comprised of a series of overlapping oligonucleotides 25 bases long. Eight sets of probes (one perfect match probe and three mismatch probes, each with one of the three mismatch nucleotides in the 13th position for the plus strand and the complementary minus strand) were generated for each of the 70,208 bases that can be determined by the microarray tiles. This design allows reporting the actual sequence of the virus in the sample even though its sequence is up to 20% divergent from the sequence the design modeled. Sequence composition of each tile is shown in S1 Table. Each of the four Ebolaviruses has tile detectors comprising 11,851 bases of the 18,959-base genomes. Multiple overlapping variable region tiles ensure that most variants will be sequenced. Conserved region tiles are important to provide sequence for computational determination of the genome identity.

The complete description of the microarray is available at ArrayExpress (https://www.ebi.ac.uk/arrayexpress/experiments/) with the accession number A-MTAB-670.

Primer design

Primers were designed to amplify approximately 500-base fragments requiring multiple overlapping amplicons to cover the larger tile detectors. Primer pairs were assembled into 4 pools

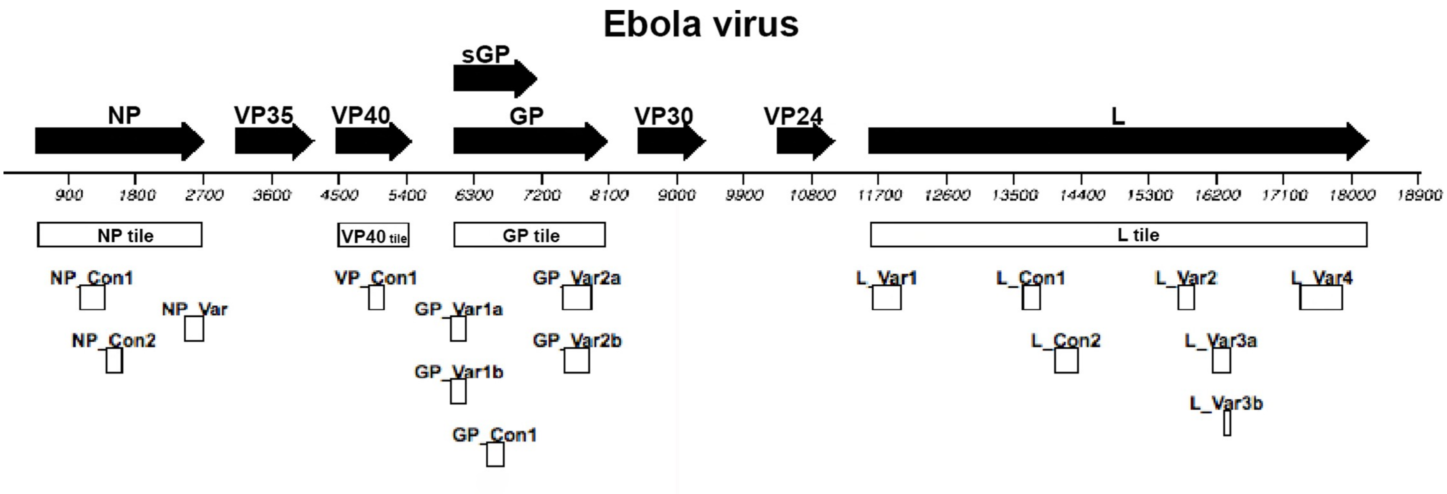

Fig 1. Microarray tile design. The full genome of Ebolavirus is indicated by the black line with bases numbered. The filled black arrows indicate the coding sequences for the eight virus proteins. Genes encode nucleoprotein (NP), nucleocapsid-associated (VP24) viral proteins (VP35, VP30), matrix protein (VP40), and RNA polymerase containing Large protein (L). The glycoprotein (GP) gene encodes four proteins, resulting from RNA editing and proteolytic cleavage: transmembrane $GP_{1,2}$, secreted glycoprotein (sGP and ssGP), and the delta peptide which is the C-terminal cleavage product of the sGP precursor. The white boxes indicate the location of the sequences that are covered by the overlapping probes called "tiles" on the microarray. Conserved sequence tiles (Con) and variable sequence tiles (Var) are indicated as the smaller boxes below the full coding sequence tile in the position of the sequence covered. There are separate sets of tiles for Ebola virus, Bundibugyo virus, Taï Forest virus, and Sudan virus (shown in S1 Fig). There are 83 tiles over the four evaluated Ebolaviruses, covering 11,842–11,850 bases (~62%) of the genome depending on the Ebolavirus.

https://doi.org/10.1371/journal.pone.0263732.g001

to achieve multiplex amplification. Ebolaviruses divergence required the design of 2 sets of 4 primer pools: one set for use with BDBV/TAFV, and one set for use with EBOV/SUDV. To achieve sequencing of the cultured recombinant virus, rVSV-EBOVgp-GFP [25], two additional primers were added to the EBOV pool (S2 Table) because the sequence flanking the EBOVgp coding sequence was different from the flanking sequence in the native EBOV genome. Primer pairs were tested in single-plex PCR procedures using genomic RNA templates. Sensitive and specific primers were selected and optimized into four mixtures, using Oligo 7 software (Colorado Springs, CO) to avoid dimer formation, false priming, and internal hairpins. The performance of the primers was visualized using a sensitive capillary electrophoresis system (2100 Bioanalyzer Instrument, Agilent Technologies, Inc., Santa Clara, CA). The primer sequences and the pool to which each belongs are listed in S2 Table.

Source of Ebolavirus and nucleic acid extraction

Samples of the four Ebolaviruses were obtained from BEI Resources (Manassas, VA, BEIRESOURCES.ORG). For EBOV isolate Mayinga (NR-31806) and Bundibugyo virus (NR-31812), reference materials were obtained as previously isolated RNA extracts, as described [26]. For Taï Forest virus (NR-44241), Sudan virus (NR-31810), and EBOV variant Makona (NR-49462), irradiated whole cell extracts containing inactivated virions were obtained from BEI Resources. Ebolavirus genomic RNA was isolated from these with the QIAamp® MinElute® Virus Spin Kit (Qiagen; cat no.57704) per the manufacture's instruction with the modification that the incubation/extraction step was extended to 1 hour for increased yield.

Multiplex reverse transcription PCR

RNA extracts were measured for quality control in the Nanodrop Spectrophotometer (ND-1000, Thermo Fisher Scientific, Wilmington, DE). Processing followed the previous method generally [18] with the following adaptations. In a clean cabinet, an annealing mix of 4 μL

DEPC water, 1 μL of 40 μM random ninemer, 1 μL 10mM dNTPs, and 1 μL each of the internal control templates (1 pg/μL) designed from the sequence of *Arabidopsis* genes NAC1 and TIM [27] were added to 4 μL ($4X10^5$ genomic copies) of ebolavirus RNA. The reaction components were mixed gently and placed in a thermocycler 5 min at 65˚C, 5 min at 4˚C. A Reverse Transcription mix of 2 μL SuperScript™ III (200 U/μL) Reverse Transcriptase (Thermo Fisher Scientific, South San Francisco, CA), 8 μL 5X First strand buffer, 4 μL DTT (25mM), and 2 μL of RNAsin (40 U/μL) was prepared and added to the annealing mixture, mixed gently and then heated in a thermocycler 1 min at 25˚C, 60 min at 50˚C, 15 min at 75˚C. A PCR master mix was prepared by adding 100 μL DEPC water, 40 μL 5X GoTaq G2 Buffer, 24 μL $MgCl_2$ (25 mM), 4 μL dNTP (10 mM), and 8 μL GoTaq G2 5 U/μL (Promega). Forty-four ul of this mix was added to each of 4 pools, along with 1 μL primer mix per pool (See S2 Table for composition of primers in each pool). RT product (5 μL) was added to each pool, mixed gently and heated 1 cycle of: 5 min at 24˚C, 2 min at 94˚C, 11 cycles of: 30 sec at 94˚C, 30 sec at 50˚C, the lower temperature increasing 1˚ per cycle to 68˚C then 29 cycles of: 30 sec at 94˚C, 120 sec at 60˚C. After the thermocycling, the samples can be frozen at -20˚ or advanced to the microarray processing steps.

Microarray hybridization and sequence analysis

Microarray hybridization, processing and scanning were carried out following the previously published method using a GeneChip assay kit (Affymetrix, Inc.) [28]. Sequence data were generated as base calls using the Gene Chip Sequence Analysis Software (GSEQ, Affymetrix, Inc), which generates a FASTA file containing the sequence of each of the tiles on the microarray. The details of microarray results including the image file (CEL files), base-calling file (CHP) and the final called base sequence (txt files) of each microarray are available at ArrayExpress (https://www.ebi.ac.uk/arrayexpress/) under the accession number E-MTAB-10007 for the Ebola genome resequencing.

Sequence data in the FASTA file is processed by a custom data analysis pipeline, *ebola_i2o*. A very simple Graphic User Interface allows choice of the pipeline and choice of the FASTA file. With a click on the "run" button, the *ebola_i2o* executes a series of PERL-based scripts that link together some minor calculations and open source software: BLAST for database searching [29], MUSCLE for multiple sequence alignment [30], Phyml for creating phylogenetic trees [31] and Newick Utilities for processing phylogenetic trees [32], all unseen by the operator. The *ebola_i2o* pipeline is loaded on a Dell Precision Tower 7910 workstation with Dual Intel Xeon Processor E5-2 650 v3, 64GB memory and two hard drives (1TB and 0.5TB). As a first step the pipeline creates a consensus sequence (determined by plurality) for each of the 52 short tiles. As an example, the nucleoprotein (NP) full length tile overlaps NP Con1. The pipeline compares the two tiles in the area where they overlap and modifies the NP Con1 to have the consensus (determined by plurality) base at each position. Next, a C3 score is calculated for each tile sequence. The C3 score is the total number of nucleotides that occur in runs of three or more consecutive (non-N) base calls, expressed as a percentage of the length of the tile sequence (in bases) [20]. The C3 score is a measure of the quantity of hybridized DNA and the quality of hybridization. Tile sequences with C3 scores greater than or equal to 20.0 are considered to represent true positive hybridization events; tile sequences with lower scores are postulated to be products of cross-hybridization or assay noise.

Tile sequences, including the 6 designed to detect Marburg virus, are searched for homologous sequences in an in-house Filovirus specific database, FilovirDB, that is stored on the Dell Precision Tower 7910 workstation, using the BLAST alignment algorithm. BLAST results for each tile: the top three database hits including their Bit-score, E-value, accession number and

scientific name are stored in a BLAST report along with the C3 score for each tile. This BLAST Report is deposited into a date and time stamped folder. A text file with the original sequence report of all the tiles is also added to the folder.

We have identified 13 short segments in each of the genomes of the four human-infecting Ebolaviruses (six conserved regions and seven variable regions, see Fig 1) whose consensus sequences taken together allow us to accurately place a test Ebolavirus in a phylogenetic tree of Ebolaviruses of known strain type with minimal computational time. Unseen by the operator, the pipeline compares these 13 segments and chooses, among the 4 Ebolaviruses, the tile with the highest C3 score, most likely to represent a real hybridization event of a tile with its cognate DNA sequences. Each high-scoring tile sequence is then aligned to the reference alignment file of the corresponding Ebolavirus. This reference alignment file stores the alignment of 160 representative ebolavirus sequences for the given genome segment. Alignments are performed in MUSCLE software [30]. This process is repeated for all 13 short segments (tile + reference sequences), which are subsequently concatenated into a single master alignment that the automatic pipeline uses to create a phylogenetic tree with the method of maximum likelihood and General Time Reversible (GTR) model of substitution, which assumes different rates of substitutions and frequencies of occurrence of nucleotides. The pipeline performs phylogenetic analysis in PhyML software [31], an example of the tree output of the pipeline is shown in S2 Fig, displaying the tree as seen in SeaView [33]. The phylogenetic tree output files are loaded in the folder along with the BLAST Report.

The code for the ebola_i2o pipeline and construction of FilovirDB is freely available at: https://github.com/openbox-bio/ebola_i2o.

To obtain a consensus Ebolavirus-RMA sequence, the FASTA files from the results of 3 independent microarray procedures per nucleic acid sample can be loaded into one of many alignment programs. In our analyses, we used the software package, Sequencher (Gene Codes Corp, Ann Arbor, MI), to align the tile sequences to the reference sequence identified as the closest match in the phylogenetic tree constructed by the pipeline. We generated a final consensus sequence by plurality rule for each sample. A graphic outline of the full analysis pipeline is shown in Fig 2.

Though no Marburg virus nucleic acid was obtained to test the full RMA process, mock FASTA files were constructed by inserting the Marburg virus sequence selected for the 6 Marburg tiles into an Ebolavirus-RMA output file with only non-called bases (Ns) in the other tiles. These FASTA files were loaded into the pipeline which assessed the quality of the tile sequences then searched them against the FilovirDB resulting in output files identifying the samples as Marburg virus, demonstrating the performance of the pipeline with the Marburg virus tile sequences.

Next generation sequencing (NGS)

Library preparation and HiSeq system sequencing were performed following established laboratory protocols [34], which are summarized below. RNA samples were processed following the protocol for the Illumina TruSeq Stranded mRNA Sample Preparation Kit. One microgram of total RNA was fragmented and reverse-transcribed into cDNAs (the poly(A)-tailed RNA enrichment was skipped, as viral RNA does not contain poly(A) tails). Double-strand cDNAs were adenylated at the 3' ends and individually indexed, followed by limited-cycle (15) amplification. Paired-end sequencing (100x2 cycles) of multiplexed RNA samples per lane was carried out on an HiSeq 2500 sequencer (Illumina, Inc., San Diego, CA). Fastq files of sequence reads were assembled to the appropriate reference with FDA CBER's High-performance Integrated Virtual Environment (HIVE) Hexagon aligner [35], a cloud-based environment

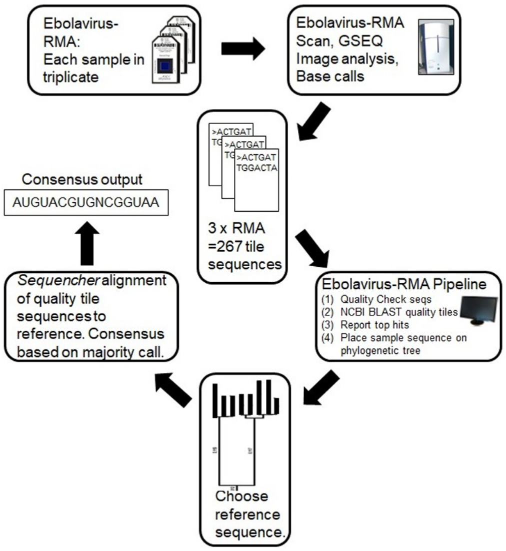

Fig 2. Flow chart of processing and analysis of Ebolavirus-RMA chip results. After hybridization and washing, Ebolavirus-RMA chips (N = 3 per RNA sample) are scanned and the output is generated in the form of FASTA files with the base calls in the tiles where enough hybridization occurred, corresponding to the region of one of the 4 Ebolavirus genomes. After quality filtering, the tile sequences above cutoff are searched against an in-house Filovirus specific database, FilovirDB, to identify the most similar sequence files. The combined sequences are also aligned against reference Ebola sequences, allowing the sample to be phylogenetically characterized to select the closest matching reference sequence. The reference sequence and original three FASTA files are then loaded into an alignment software and a consensus of the three Ebolavirus-RMA chips is generated.

https://doi.org/10.1371/journal.pone.0263732.g002

optimized for storage and analysis of extra-large sequence data. The Hexagon alignment was further processed with the HIVE-heptagon sequence profiling tool [36] which formulates a consensus from the base with highest frequency over all reads at each position.

The assembled consensus NGS sequence for an RNA sample was compared to the Ebolavirus-RMA sequence for the same sample with ClustalW operated within the MacVector software (version 17.5.5, MacVector, Inc, Apex, NC). ClustalW parameters were Slow pairwise alignment mode, open gap penalty = 15.0, extended gap penalty = 6.7, delay divergent = 30%, transitions: weighted. The raw Illumina NGS reads and the assembled consensus sequence are available at ArrayExpress (https://www.ebi.ac.uk/arrayexpress/) with the accession number E-MTAB-10102.

rVSV-EBOVgp-GFP antibody neutralization and non-neutralization assay

Replication-competent recombinant vesicular stomatitis virus (VSV) constructs in which the VSV-G envelope protein was replaced with the EBOV glycoprotein (GP), followed by the green fluorescence protein (GFP) gene, termed rVSV-EBOVgp-GFP, were prepared as described previously [25]. Prior to the neutralization experiment, a stock culture of rVSV-EBOVgp-GFP was RNA extracted and six replicate Ebolavirus-RMA chips were processed to produce an RMA consensus of the sequence of the EBOVgp coding sequence. The details of microarray results including the image file (CEL files), base-calling file (CHP) and the final called base sequence (txt files) of each microarray are available at ArrayExpress (https://www.ebi.ac.uk/arrayexpress/) under the accession number E-MTAB-10008 for the resequencing of the Ebola glycoprotein gene inserted into Vesicular Stomatitis Virus. The initial stock RNA was also subject to Illumina next generation sequencing for comparison to the Ebolavirus-RMA consensus sequence. The raw reads and the assembled consensus sequence are available at ArrayExpress (https://www.ebi.ac.uk/arrayexpress/) with the accession number E-MTAB-10157.

To monitor genome changes, African green monkey kidney (Vero E6) cells were infected with rVSV-EBOVgp-GFP as described in previous neutralization assays [25, 37]. Briefly, the EBOV neutralizing human monoclonal antibody (mAb) KZ52 (Integrated BioTherapeutics, Inc.) [38] was incubated at a concentration of 2μg of antibody in 100μl of culture medium or PBS as control with rVSV-EBOVgp-GFP. After 1 hour, the mixtures were added to Vero E6 cells in a 6-well plate. Four days post infection, culture supernatant was recovered for sequencing (Round 1) and 100μl passaged onto fresh Vero E6 cells or treated with 2μg of KZ52, then added to fresh Vero E6 cells. Three days post passage, supernatant was collected as Round 2 and passaged onto fresh Vero E6 cells with the same treatments. Three days post passage, supernatant was collected as Round 3. During all 3 rounds of passage, mAb KZ52 was present in one culture and a parallel culture received PBS control. RNA extraction of each Round of culture supernatant was sequenced by the Ebolavirus-RMA to reveal the sequence of the EBOV glycoprotein. The Round 3 RNA sample was also submitted to Illumina HiSeq NGS and the EBOV glycoprotein assembled for comparison with the RMA result. The raw reads and the assembled consensus sequence are also available at ArrayExpress under the accession number E-MTAB-10157.

ClustalW multiple sequence alignment

Nucleotide sequences were aligned in the software *MacVector* (MacVector, Inc., Apex, NC) using the ClustalW algorithm according to the instructions in the MacVector User Guide with default parameters.

Results

Design of the Ebolavirus-RMA

The Ebolavirus-RMA was designed to determine the genome sequence of the four Ebolaviruses that pose a threat to human populations. Additional human non-pathogenic Ebolavirues such as Reston virus and Bombali virus were not included to reserve space for coverage of the sequence diversity within the human-infecting viruses. Each virus sequence is represented by 17 to 28 locations on the microarray depending on the Ebolavirus (Figs 1 and S1). The locations, called tile detectors, are prototype sequences that fully cover 4 of the 7 genes in the ebolavirus genome. Prototype or reference sequences were selected based on the consensus of all ebolavirus NCBI database entries prior to the 2013–2016 epidemic. Due to the extent of divergence between the 4 Ebolaviruses, a complete set of tile detectors was designed for each Ebolavirus to ensure that high-accuracy sequence results would be obtained (S1 Fig).

The target genes were selected based on an assessment of which genes have shown variability, demonstrated a role in virulence, potential impact on diagnostics or therapeutics, and are significant for monitoring genetic drift in outbreaks. For example, genetic drift in matrix protein (VP40) and GP [39] as constituents of the envelope of the ebolavirus virion are more likely to affect immune system recognition of the virus than other genes [40]. The virus RNA Dependent RNA polymerase encoded in the L gene and the nucleoprotein (NP) gene, as a consequence of their roles in replicating and coating the single strand-RNA genome respectively, may affect the mutability of the virus and were among the genes that contained unique nucleotide variants in the Makona strains [41]. Most of the assays used for detection and diagnosis of Ebolavirus in the field are currently using either serological or RT-PCR-based nucleic acid tests (NAT) [42, 43] that target these 4 genes.

For target regions that are highly variable, multiple tiles were designed for the same sequence region to cover the sequence divergence of the variants (Fig 1, labeled "Var"). To aid in the database search of the output sequence of Ebolavirus, we also included overlapping tiles

covering highly conserved regions of sequence that provide anchors (Fig 1, labeled "Con"). This composition of the microarray was designed to achieve the accurate sequencing of the range of nucleotide variability among the species of Ebolavirus rather that complete genome coverage of one reference strain as in other RMAs [23].

Tiles for Marburg virus were included to detect this related Filovirus when a patient with overlapping symptoms is mistakenly diagnosed with Ebola Disease. There is not enough coverage nor an intent to sequence the Marburg virus genome.

Evaluating the Ebolavirus-RMA with repository ebolavirus material

We initially tested the ebolavirus protocol with repository samples of extracted virus RNA or irradiated crude infected Vero cell lysate obtained from BEI Resources (Table 1). With a starting input of $4.0x10^5$ copies of viral RNA, base call rates of 98.8–99.6% were obtained for 4 of the 5 Ebolaviruses or variants tested (Table 1). Call rates were lower only for the SUDV strain Boniface, possibly due to specimen matrix interference or divergence from the tile designs.

To assess the accuracy of the sequence generated by the Ebolavirus-RMA and the accuracy of the pipeline in assigning a correct reference sequence, we compared the generated consensus sequence with the sequence file retrieved from GenBank for the accession number provided by the sample supplier. For all three Ebolaviruses and two EBOV variants, a level of agreement of >98% was obtained for 4 of 5 with only SUDV, which had an inferior call rate, having lower (Table 1). Furthermore, our custom-analysis pipeline placed the sequences derived by the microarray on the phylogenetic tree adjacent to the sequences of the repository Ebolaviruses and variants (S2 Fig). To condense the phylogenetic tree display of the results from all the Ebolaviruses, an alignment was performed separately from the pipeline using the consensus sequence from three microarray outputs for each RNA sample and the full-length ebolavirus genomes from the database (Fig 3). Notably, the algorithm placed the EBOV Mayinga isolate (AY142960) and Makona (KJ660347.2) variant, which differ by only 2.9% across their genome sequence, on separate branches of this tree. Therefore, the microarray obtains an accurate sequence, and the pipeline is a suitable way to automate the interpretation of the FASTA outputs and assign the sample the correct identity even among samples that have a similar sequence.

To compare the results of the Ebolavirus-RMA with another leading sequencing platform, we chose EBOV Mayinga isolate as an example, prepared NGS libraries from the RNA sample, processing them on an Illumina sequencer. We obtained full coverage of the 19kb EBOV

Table 1. Microarray sequencing results for repository ebolavirus samples.

Source Sequence Accession	Ebolaviruses	Base Calls	Non-Calls	Total	% Called	RMA compared to source file		differences from NGS (bases)	% agreement[c]
						Variant bases	% called correctly[b]		
AY142960	Ebola virus Mayinga	11762	88	11,850	99.3	10	99.2	10	99.20
KU182911.1	Bundibugyo	11703	142	11,845	98.8	3	98.8	NA	NA
FJ217162.1	Taï Forest	11800	42	11,842	99.6	5	99.6	NA	NA
FJ968794.1	Sudan	10727	1117	11,844	90.57	71	89.97	NA	NA
KJ660347.2	Ebola virus, Makona[a]	11720	130	11,850	98.90	7	98.84	NA	NA

[a]Genome sequence derived from the Guinea sample is described as the Makona variant.

[b]Counting all microarray bases aligning with the reference sequence.

[c]Counting all microarray bases aligning with the NGS sequence.

https://doi.org/10.1371/journal.pone.0263732.t001

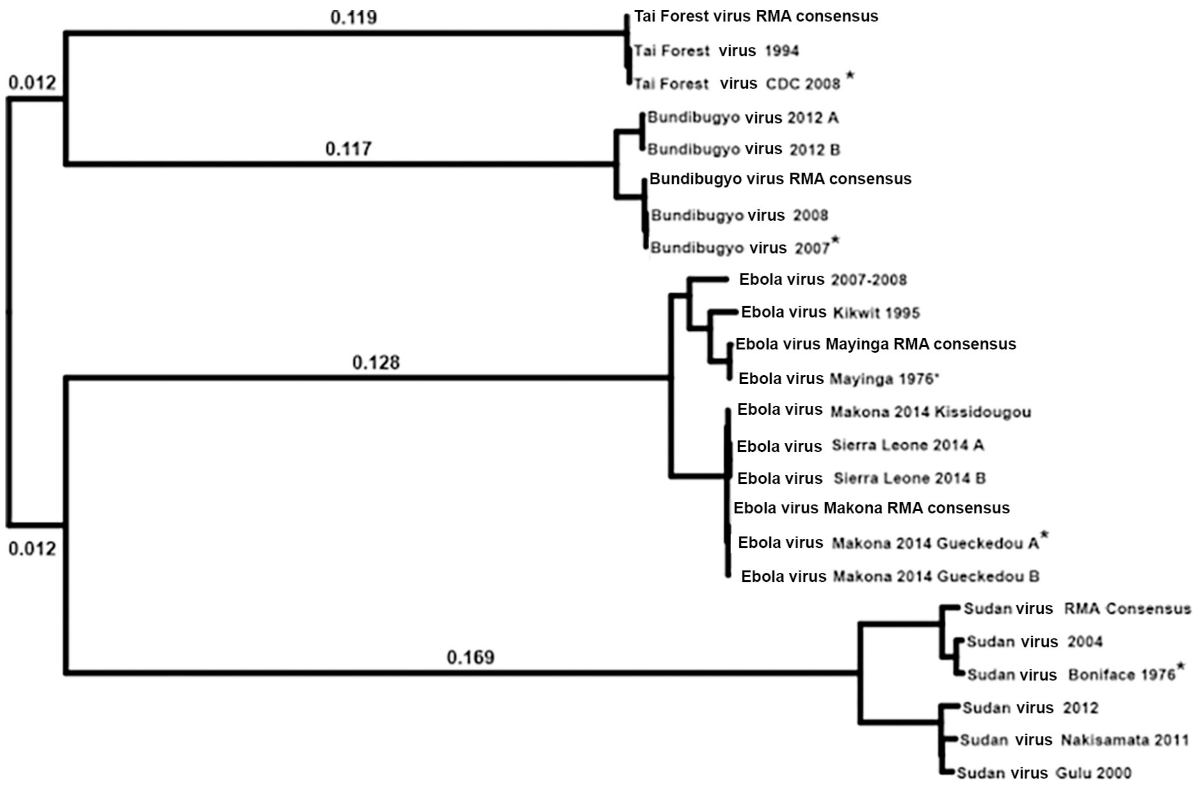

Fig 3. Phylogenetic tree alignments. A phylogenetic tree of Ebolavirus-RMA results for five repository RNA samples consensus sequence results (three replicates for each sample) aligned to representative database entries for five Ebolaviruses/variants. Alignments are done with MUSCLE software using default parameters in MacVector version 17.0 with the bootstrap method. The asterisks (*) denote the database sequence file for the repository Ebolavirus RNA samples that were processed by the Ebolavirus-RMA. Numbers on the branches are distances in phylogenetic units.

https://doi.org/10.1371/journal.pone.0263732.g003

genome at a read depth of ≥100,000. The RMA and NGS sequencing outputs of the same EBOV Mayinga isolate RNA sample showed only 10 base call discrepancies and 88 non-called bases in the Ebola-RMA consensus (≥99.20% agreement, Table 1). These results provide evidence that the Ebolavirus-RMA platform produces sequence that can be of value in identifying the virus strain which should be as effective in outbreak tracing as standard NGS methods with much simpler procedures.

Table 2. Ebolavirus-RMA response to ebolavirus quantity. Ebola virus Mayinga isolate (NR-31806, BEI Resources) RNA quantity was serially diluted ten-fold.

Input Quantity: Genome Copies	Sequence Quality: Weighted Average C3 Score[a]	BLAST Report Result
40	28.31	EBOV[b]
400	33.71	
40,000	57.34	
400,000	97.15	

[a]Average of the C3 scores assigned to each EBOV tile that are reported (>20), weighted by length of each tile in bases.

[b]All four RMA pipeline phylogenetic tree results place the RMA sequence adjacent to EBOV sequence files with accession numbers, AF499101, AY142960*, KC242791, KC242801 (S2 Fig). *This file is the database sequence of the EBOV strain used as input RNA for these RMAs (AY142960).

https://doi.org/10.1371/journal.pone.0263732.t002

To estimate the sensitivity of the Ebolavirus-RMA for detection of Ebolavirus and evaluate whether the sequence quality decreases with decreased amounts of input RNA, we serially tenfold diluted samples of EBOV Mayinga, with the lowest dilution including 40 copies per reaction. While the call rate decreases with dilution, the output sequence and search algorithm remained able to correctly assign the sample to the appropriate reference with the lowest number of genome copies tested (Table 2). Even at 40 copies of input RNA and a weighted mean C3 score of 28 for the EBOV tiles, the Mayinga isolate could be distinguished and assigned to a reference that would serve to identify the Ebolavirus present in a clinical sample, though 400,000 copies would be required to accurately sequence the viral genome.

All the microarray data from this microarray study is available on the ArrayExpress repository (https://www.ebi.ac.uk/arrayexpress/) with the accession number E-MTAB-10007.

Ebolavirus-RMA sequencing of a replicating recombinant virus

To demonstrate that the Ebolavirus-RMA as a system could effectively monitor the genetic drift of the ebolavirus genome as might occur in outbreak scenarios, we followed a previously described approach generating escape mutants with a neutralizing antibody [44, 45]. We used the replication competent recombinant virus, rVSV-EBOVgp-GFP. The recombinant virus was engineered by molecular substitution of the coding sequence of the VSV glycoprotein with the EBOV glycoprotein in a plasmid, which, when transfected into cells, accomplishes assembly of infectious virus. The recombinant virus was designed for use in biosafety level 2 laboratory (BSL-2) neutralization assays to evaluate antibody response to Ebola virus disease and vaccination [25, 37, 46]. Previous studies have demonstrated that this virus performs similarly to wild-type Ebola in neutralization assays performed in a BSL-4 laboratory [46]. We have observed that, after multiple passages in Vero E6 cells with neutralizing antibodies, "escape mutants" evolve that are no longer blocked by the antibody. Identification of base changes in the EBOV glycoprotein coding sequence that could result in these escape phenotypes would demonstrate that the Ebolavirus-RMA would detect EBOV genomic drift. rVSV-EBOVgp-GFP was passaged in the presence or absence of a mAb, KZ52, with a known neutralizing epitope on the ebolavirus GP [47]. Virus stock was collected before passaging (initial stock) and after 1, 2 and 3 passages. In the first passage the mAb KZ52-treated virus was severely restricted in infection rate compared with the untreated virus. By the third passage, the virus in the presence of mAb KZ52 was achieving an infection rate similar to the untreated virus (Fig 4).

RNA was extracted from virus stocks at each passage and sequenced by the Ebolavirus-RMA. Only the EBOV tiles in the GP region of the microarray resulted in base calls. Results are shown in Table 3. The positions where substitutions were detected illustrate several different patterns of genomic drift. At position N(506) the Ebolavirus-RMA results, confirmed by NGS, identified an amino acid substitution known to block binding of the KZ52 antibody [48, 49] that only appears in the 3rd round of passage under pressure from the KZ52 antibody. This is a tangible example of the genomic drift that the Ebolavirus-RMA is designed to detect. Substitutions at positions Q(28), I(129) and Q(467) appear not to be responding to selective pressure because they either appear, then revert to the original sequence over the time in culture with or without pressure from the KZ52 antibody, or they appear without selective pressure. At the codon for amino acid V(662), it appears that a base designed in the plasmid used to create the recombinant virus was substituted, detected by Ebolavirus-RMA and confirmed by NGS, when the original virus emerged, changing the sequence to encode isoleucine, which remained fixed in all virus passages. At two positions, V(52) and S(422), the Ebolavirus-RMA base call was not confirmed by NGS suggesting there may be a flaw in the RT-PCR steps or microarray tile at these locations, since the incorrect base call is made with multiple different

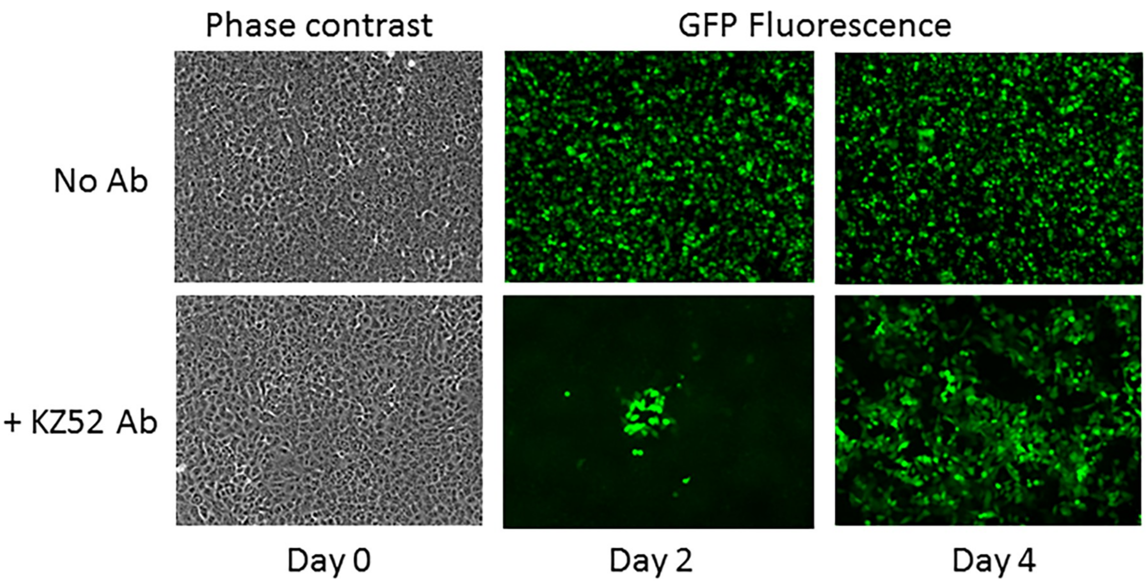

Fig 4. Micrographs of cells infected with rVSV-EBOVgp-GFP recombinant virus. Vero E6 cells are pictured on successive days in passage two. The top panel was cultured in the absence of antibody. The bottom panel was cultured in the presence of monoclonal antibody KZ52 which neutralizes virus infection. Day 0, initial infection, is shown in phase contrast to reveal the confluent Vero E6 cells. Over time, escape mutants arise as indicated by the numerous infected cells in the presence of KZ52 on day 4. Expression of GFP indicating virus replication was visualized under the fluorescent microscope at 100X magnification on day 2 and day 4.

https://doi.org/10.1371/journal.pone.0263732.g004

RNA samples. Future work will determine the source of these variants (see Discussion). However, the overall agreement between NGS sequence of the rVSV-EBOVgp-GFP recombinant virus and the Ebolavirus-RMA results for the same sample taken from the initial stock virus was 99.2% and for the RNA from the KZ52 selected round 3 virus, the agreement was 97.1%, which is comparable to the rate of agreement which was obtained with the Ebolavirus-RMA and NGS in the full-length genome samples.

All the microarray data from the replicating recombinant virus study is available at ArrayExpress (https://www.ebi.ac.uk/arrayexpress/) with the accession number E-MTAB-10008.

Table 3. Recombinant virus, rVSV-EBOVgp-GFP, point mutations identified by Ebolavirus-RMA sequencing. RNA was extracted from virus stock cultured with (selected) or without (nonselected) Ebola virus neutralizing antibody KZ52.

Sample	Sequence method	Amino Acid Substitutions Detected							% bases called
**Plasmid DNA**	**Sanger**	**Q(28)**	**V(52)**	**I(129)**	**S(422)**	**Q(467)**	**N(506)**	**V(662)**	**100**
Initial stock[a]	NGS	Q	V	I	S	Q	N	I	100
Initial stock	Ebolavirus-RMA	Q	A	I	F	Q	N	I	99.7
Nonselected Round 1	Ebolavirus-RMA	Q	A	I	X[b]	R	N	I	96.53
Nonselected Round 2	Ebolavirus-RMA	Q	A	V	F	Q	N	I	99.21
Nonselected Round 3	Ebolavirus-RMA	R	A	V	F	X	N	[I][c]	94.4
KZ52 selected Round 1	Ebolavirus-RMA	R	A	V	X	R	N	[I][c]	93.26
KZ52 selected Round 2	Ebolavirus-RMA	Q	A	X	X	X	N	[I][c]	94.2
KZ52 selected Round 3	Ebolavirus-RMA	Q	A	V	X	Q	**D**	[I][c]	97.42
KZ52 selected Round 3	NGS	Q	V	V	S	Q	**D**	I	100

[a]Virus stock.

[b]non-called bases in these codons make interpretation of the amino acid ambiguous.

[c]inadequate base calls prevent unambiguous translation. Though the base substitution that alters the codon from V to I was observed, non-called bases in other positions in the codon make the encoded amino acid uncertain.

https://doi.org/10.1371/journal.pone.0263732.t003

Out-group comparison to non-pathogenic Ebola species

To demonstrate that the Ebolavirus-RMA as a system reports a sequence that is specific to the human pathogenic Ebolavirus species, we performed *in silico* comparisons of the RMA consensus sequences for EBOVs (Mayinga and Makona), BDBV, SUDV and TAFV to the species not pathogenic to humans (Bombali ebolavirus and Reston ebolavirus). Note that the RMA contains probes to sequence 11,851 bases of the 18,959-base genomes, thus a perfect match would have 62.5% identity. These comparisons (Table 4) show that the sequence of each Ebolavirus species or isolate determined by the Ebolavirus-RMA has the highest identity to the sequence deposited in the Genbank NCBI database for the source material RNA. The identity to all other sequence files were low enough to clearly distinguish the divergent species.

Discussion

This paper describes the evaluation of the Ebolavirus-RMA as a rapid technology to obtain the critical sequence of the ebolavirus genome. The goal of a simplified method to monitor genomic drift of Ebolavirus during an outbreak was achieved as demonstrated by the sequencing of a VSV-EBOVgp recombinant virus during passage in culture under selective pressure from a neutralizing antibody. The Ebolavirus-RMA detected a base substitution responsible for an amino acid replacement known to eliminate the binding of the neutralizing antibody. The number of substitutions detected and confirmed by NGS (2 non-synonymous base substitutions) suggest a much higher nucleotide substitution rate than observed for Ebolavirus in nature, 7.06×10^{-4} substitutions/site/year [12].

Analysis of the base call output from the scanner software with a custom designed bioinformatic pipeline and alignment of three replicate outputs with the reference sequence selected by the pipeline to form the final consensus achieved a high sequencing accuracy. This workflow results in base calls over the genome sequence covered by the microarray at accuracies of 97.1% to 99.2% agreement with the reference sequence of the Ebolavirus variant. This accuracy was obtained with 4×10^5 genome copies, which is well within the average number of copies in an acutely infected patient of 2.7×10^7/mL [50]. This capability compares well to the standard NGS technologies that were deployed in the West African and Central Africa outbreaks. With Illumina HiSeq as we used for comparison to the Ebolavirus-RMA results, approximately 0.1–1% of bases are called incorrectly [51]. For a genetically homogenous sample, the effects of

Table 4. Percent sequence identity of Ebolavirus-RMA sequence outputs with GenBank NCBI database sequences for Ebolaviruses. Header row contains the name and accession number of each database file. The first column contains the name of each resequencing microarray output which is a consensus of three microarrays.

	Ebola virus Mayinga AY142960	Ebola virus Makona KJ660347.2	Bunibugyo virus KU182911.1	Tai Forest virus FJ217162.1	SUDV virus FJ968794.1	BOMBALI virus MW056492.1	BOMBALI virus MW056493.1	RESTON virus MF540570.1	RESTON virus MT796851.1
EBOVZ-RMA[a]	62	60.4	43.2	31.8	40.3	41.4	41.5	41	30.7
EBOVG-RMA[b]	60.2	61.8	42.9	31.8	40.2	41	30.1	29.8	29.9
BDBV-RMA[c]	31.6	31.6	61.8	46.3	29.5	30.1	41	40.6	41.5
TAFV-RMA[d]	31.9	31.9	34.4	62.3	29.7	30.2	41.1	40.8	41.5
SUDV-RMA[e]	26.9	26.8	27	37.1	56.4	26.3	36.2	37.3	38

[a]Ebola virus Mayinga,

[b]Ebola virus Makona,

[c]Bunibugyo virus,

[d]Tai Forest virus,

[e]Sudan virus.

https://doi.org/10.1371/journal.pone.0263732.t004

these base miscalls can be mitigated by establishing a consensus sequence from high-coverage sequencing reads [51]. The Oxford Nanopore MinION has been used in EBOV sequencing and has attributes of portability and lower cost than standard NGS. However, the large number of sequence reads require powerful bioinformatic tools and expertise to successfully assemble an ebolavirus genome. In contrast, we have generated a consensus sequence from three microarray results to achieve the level of accuracy described here. The automated pipeline performs downstream bioinformatics processing with little more that the click of a button on the graphic user interface. The time between raw data acquisition and final sequence output, aligned to a reference ebolavirus sequence can be within the same day. The time between initiation of the workflow with an RNA sample and final sequence was 26 hours with the BEI Resource provided specimens. This capability could be enabled in an endemic country and would have established the identification of Ebola virus in the DRC outbreak facilitating the use of the rVSV-ZEBOV-GP vaccine or establishing the genomic identity between the isolates earlier this year in Guinea and the Makona strain thus pointing to the resurgence of the outbreak.

Below concentrations of 10^5 RNA copies or in specimens like the Sudan virus, the base call rate was lower so that the platform was no longer able to produce the detailed genome sequence. However, error rates were not affected, and the sequence results obtained were sufficient for identification of the virus infection, which means that the assay has applicability in real-time public health surveillance efforts. Another possible flaw in the system was revealed when Ebolavirus-RMA sequence of the rVSV-EBOVgp-GFP recombinant virus was compared with NGS of the same RNA sample. Though further work is needed to characterize this flaw, the error only occurred when RNA encoding EBOV GP was derived from the recombinant virus. The same codons in RNA from five different Ebolavirus full-length genomes were base-called correctly by the Ebolavirus-RMA. Future work will re-design this region of the microarray and design primers that are specific for divergent strains like Sudan. We hypothesize that the higher sequence divergence of the Sudan virus from the other 3 Ebolaviruses makes some of the primer pairs unsuitable for amplification and the genome of the sample may be divergent enough from the tile detector sequences to make hybridization inefficient. In future work the primer pools will be further optimized, and additional variant tiles added to the array to improve sequencing of the Sudan virus.

In summary, comprehensive genome sequencing of whole virus samples and the passage-specific variants detected in the cultured recombinant virus indicate the Ebolavirus-RMA is suited for further evaluation with ebolavirus samples toward acceptance as a tool to evaluate clinical specimens.

Supporting information

S1 Table. The sequences of all the tiles on the Ebolavirus-RMA. Tile Sequences are in a Microsoft Excel spreadsheet.
(XLSX)

S2 Table. Sequences of all primers used to amplify the nucleic acid samples. Oligonucleotide primer sequences are in a table in a Microsoft Word document.
(DOC)

S1 Fig. Tile diagram for all four Ebolaviruses. The genome and the eight coding sequences of Ebolaviruses are shown at the top. Beneath and aligned with the sequence content, are the tiles for each Ebolavirus. Note that greater variability among Sudan ebolavirus genomes required additional tiles, each with slightly different sequence composition. The microarray also has six tiles in the NP region of Marburg virus that are intended to detect a related

Filovirus that may be the causative agent of disease symptoms in Ebola-negative patients.
(TIF)

S2 Fig. Phylogenetic tree pipeline output. An RNA sample of EBOV Mayinga isolate (BEI Resources, NR-31806), sequenced with the Ebolavirus-RMA (chip #EBOVMPX004). The sequence output was processed with the *ebola_i2o* pipeline. The phylogenetic tree output of the pipeline is visualized with SeaView Version 4.7 [33]. Each file in the tree carries the name assigned to that file in the database, FilovirDB. According to this tree, the genomes in FilovirDB most closely related to the Ebolavirus-RMA output sequence are KC242791, KC24801, AF499101 and AY142960. AY142960 is the database sequence entry for the BEI Resources NR-31806 sample. An arrow indicates the location on the tree of the RMA output sequence. This figure truncates the list of Ebola virus Makona sequences because the large number of sequences in the database from the 2014–2016 West African outbreak, all very similar to each other, are not necessary to display in this figure to demonstrate their phylogenetic relationship. Horizontal bar indicates the branch length equal to 0.1 phylogenetic units (approximately 10% difference).
(TIF)

S1 File.
(DOCX)

Acknowledgments

We acknowledge and thank the BEI Resources as the source of the Ebolavirus material listed above. We appreciate the contribution of Keith Peden and David McGivern for reading and commenting on the manuscript.

RD is the guarantor of this work and, as such, had full access to all of the data in the study and takes responsibility for the integrity of the data and the accuracy of the data analysis.

Disclaimer: Our comments/contributions are an informal communication and represent our own best judgement. These comments do not bind or obligate FDA.

Author Contributions

Conceptualization: Moussa Kourout, Bryan Lanning, Anjan Purkayastha, Robert Duncan.

Formal analysis: Irina Tiper, Moussa Kourout, Bryan Lanning, Robert Duncan.

Funding acquisition: Robert Duncan.

Investigation: Irina Tiper, Moussa Kourout, Bryan Lanning, Carolyn Fisher, Krishnamurthy Konduru.

Project administration: Robert Duncan.

Software: Anjan Purkayastha.

Supervision: Gerardo Kaplan.

Writing – original draft: Bryan Lanning, Robert Duncan.

Writing – review & editing: Irina Tiper, Bryan Lanning, Carolyn Fisher, Krishnamurthy Konduru, Robert Duncan.

References

1. WHO. Ebola virus disease 2021 [cited 5/25/2021]. Available from: https://www.who.int/health-topics/ebola/#tab=tab_1.

2. Kuhn JH, Adachi T, Adhikari NKJ, Arribas JR, Bah IE, Bausch DG, et al. New filovirus disease classification and nomenclature. Nat Rev Microbiol. 2019; 17(5):261–3. Epub 2019/03/31. https://doi.org/10.1038/s41579-019-0187-4 PMID: 30926957; PubMed Central PMCID: PMC6637750.
3. Jacob ST, Crozier I, Fischer WA 2nd, Hewlett A, Kraft CS, Vega MA, et al. Ebola virus disease. Nat Rev Dis Primers. 2020; 6(1):13. Epub 2020/02/23. https://doi.org/10.1038/s41572-020-0147-3 PMID: 32080199; PubMed Central PMCID: PMC7223853.
4. WHO. Ebola Situation Report: World Health Organization; 2016 [updated 28-February-2016; cited 25-May-2021]. Available from: http://apps.who.int/ebola/ebola-situation-reports.
5. WHO. Heroes of the Ebola response: World Health Organization; 2020 [updated 7 April 2020; cited 25 May, 2021]. Available from: https://www.who.int/emergencies/diseases/ebola/drc-2019.
6. Henao-Restrepo AM, Camacho A, Longini IM, Watson CH, Edmunds WJ, Egger M, et al. Efficacy and effectiveness of an rVSV-vectored vaccine in preventing Ebola virus disease: final results from the Guinea ring vaccination, open-label, cluster-randomised trial (Ebola Ca Suffit!). Lancet. 2017; 389 (10068):505–18. https://doi.org/10.1016/S0140-6736(16)32621-6 PMID: 28017403; PubMed Central PMCID: PMC5364328.
7. WHO. 11th Ebola outbreak in the Democratic Republic of the Congo declared over World Health Organization2020 [updated 18 November 2020; cited 25 May, 2021]. Available from: https://www.afro.who.int/news/11th-ebola-outbreak-democratic-republic-congo-declared-over.
8. WHO. Ebola virus disease–Guinea 2021 [updated 17 February 2021; cited 25 May 2021]. Available from: https://www.who.int/emergencies/disease-outbreak-news/item/2021-DON312.
9. Faye M. Guinea 2021 EBOV outbreak: Virological.org; 2021 [updated 12 March 2021; cited 25 May 2021]. Available from: https://virological.org/t/guinea-2021-ebov-outbreak/643.
10. Keita A, Dux A, Diallo H, Calvignac-Spencer S, Sow MS, Keita MB, et al. Resurgence of Ebola virus in Guinea after 5 years calls for careful attention to survivors without creating further stigmatization: Virological.org; 2021 [cited 25 May 2021]. Available from: https://virological.org/t/guinea-2021-ebov-genomes/651.
11. Holmes EC, Dudas G, Rambaut A, Andersen KG. The evolution of Ebola virus: Insights from the 2013–2016 epidemic. Nature. 2016; 538(7624):193–200. https://doi.org/10.1038/nature19790 PMID: 27734858; PubMed Central PMCID: PMC5580494.
12. Carroll SA, Towner JS, Sealy TK, McMullan LK, Khristova ML, Burt FJ, et al. Molecular evolution of viruses of the family Filoviridae based on 97 whole-genome sequences. J Virol. 2013; 87(5):2608–16. Epub 2012/12/21. https://doi.org/10.1128/JVI.03118-12 PMID: 23255795; PubMed Central PMCID: PMC3571414.
13. Hoenen T, Safronetz D, Groseth A, Wollenberg KR, Koita OA, Diarra B, et al. Virology. Mutation rate and genotype variation of Ebola virus from Mali case sequences. Science. 2015; 348(6230):117–9. https://doi.org/10.1126/science.aaa5646 PMID: 25814067.
14. Liu SQ, Deng CL, Yuan ZM, Rayner S, Zhang B. Identifying the pattern of molecular evolution for Zaire ebolavirus in the 2014 outbreak in West Africa. Infect Genet Evol. 2015; 32:51–9. Epub 2015/03/10. https://doi.org/10.1016/j.meegid.2015.02.024 PMID: 25745889.
15. Marzi A, Chadinah S, Haddock E, Feldmann F, Arndt N, Martellaro C, et al. Recently Identified Mutations in the Ebola Virus-Makona Genome Do Not Alter Pathogenicity in Animal Models. Cell Rep. 2018; 23(6):1806–16. https://doi.org/10.1016/j.celrep.2018.04.027 PMID: 29742435; PubMed Central PMCID: PMC5969531.
16. Mbala-Kingebeni P, Villabona-Arenas CJ, Vidal N, Likofata J, Nsio-Mbeta J, Makiala-Mandanda S, et al. Rapid Confirmation of the Zaire Ebola Virus in the Outbreak of the Equateur Province in the Democratic Republic of Congo: Implications for Public Health Interventions. Clin Infect Dis. 2019; 68(2):330–3. https://doi.org/10.1093/cid/ciy527 PMID: 29961823.
17. Lin B, Malanoski AP, Wang Z, Blaney KM, Long NC, Meador CE, et al. Universal detection and identification of avian influenza virus by use of resequencing microarrays. J Clin Microbiol. 2009; 47(4):988–93. Epub 2009/03/13. doi: JCM.01346-08 [pii] https://doi.org/10.1128/JCM.01346-08 PMID: 19279171; PubMed Central PMCID: PMC2668298.
18. Lin B, Wang Z, Vora GJ, Thornton JA, Schnur JM, Thach DC, et al. Broad-spectrum respiratory tract pathogen identification using resequencing DNA microarrays. Genome Res. 2006; 16(4):527–35. https://doi.org/10.1101/gr.4337206 PMID: 16481660; PubMed Central PMCID: PMC1457032.
19. Dalma-Weiszhausz DD, Warrington J, Tanimoto EY, Miyada CG. The affymetrix GeneChip platform: an overview. Methods Enzymol. 2006; 410:3–28. https://doi.org/10.1016/S0076-6879(06)10001-4 PMID: 16938544.
20. Metzgar D, Myers CA, Russell KL, Faix D, Blair PJ, Brown J, et al. Single assay for simultaneous detection and differential identification of human and avian influenza virus types, subtypes, and emergent variants. PLoS One. 2010; 5(2):e8995. https://doi.org/10.1371/journal.pone.0008995 PMID: 20140251; PubMed Central PMCID: PMC2815781.

21. Berthet N, Paulous S, Coffey LL, Frenkiel MP, Moltini I, Tran C, et al. Resequencing microarray method for molecular diagnosis of human arboviral diseases. J Clin Virol. 2013; 56(3):322–7. https://doi.org/10.1016/j.jcv.2012.10.022 PMID: 23219893.

22. Sulaiman IM, Tang K, Osborne J, Sammons S, Wohlhueter RM. GeneChip resequencing of the smallpox virus genome can identify novel strains: a biodefense application. J Clin Microbiol. 2007; 45(2):358–63. https://doi.org/10.1128/JCM.01848-06 PMID: 17182757; PubMed Central PMCID: PMC1829075.

23. Hardick J, Woelfel R, Gardner W, Ibrahim S. Sequencing ebola and marburg viruses genomes using microarrays. J Med Virol. 2016; 88(8):1303–8. https://doi.org/10.1002/jmv.24487 PMID: 26822839.

24. Lin B, Blaney KM, Malanoski AP, Ligler AG, Schnur JM, Metzgar D, et al. Using a resequencing microarray as a multiple respiratory pathogen detection assay. J Clin Microbiol. 2007; 45(2):443–52. https://doi.org/10.1128/JCM.01870-06 PMID: 17135438; PubMed Central PMCID: PMC1829030.

25. Konduru K, Shurtleff AC, Bradfute SB, Nakamura S, Bavari S, Kaplan G. Ebolavirus Glycoprotein Fc Fusion Protein Protects Guinea Pigs against Lethal Challenge. PLoS One. 2016; 11(9):e0162446. https://doi.org/10.1371/journal.pone.0162446 PMID: 27622456; PubMed Central PMCID: PMC5021345.

26. Towner JS, Sealy TK, Ksiazek TG, Nichol ST. High-throughput molecular detection of hemorrhagic fever virus threats with applications for outbreak settings. J Infect Dis. 2007; 196 Suppl 2:S205–12. https://doi.org/10.1086/520601 PMID: 17940951.

27. Wang HY, Malek RL, Kwitek AE, Greene AS, Luu TV, Behbahani B, et al. Assessing unmodified 70-mer oligonucleotide probe performance on glass-slide microarrays. Genome Biol. 2003; 4(1):R5. Epub 2003/01/24. https://doi.org/10.1186/gb-2003-4-1-r5 PMID: 12540297; PubMed Central PMCID: PMC151289.

28. Kourout M, Fisher C, Purkayastha A, Tibbetts C, Winkelman V, Williamson P, et al. Multiplex detection and identification of viral, bacterial, and protozoan pathogens in human blood and plasma using a high-density resequencing pathogen microarray platform. Transfusion. 2016; 56(6 Pt 2):1537–47. https://doi.org/10.1111/trf.13524 PMID: 26932359.

29. Altschul SF, Gish W, Miller W, Myers EW, Lipman DJ. Basic local alignment search tool. J Mol Biol. 1990; 215(3):403–10. Epub 1990/10/05. https://doi.org/10.1016/S0022-2836(05)80360-2 PMID: 2231712

30. Edgar RC. MUSCLE: multiple sequence alignment with high accuracy and high throughput. Nucleic Acids Res. 2004; 32(5):1792–7. Epub 2004/03/23. https://doi.org/10.1093/nar/gkh340 PMID: 15034147; PubMed Central PMCID: PMC390337.

31. Guindon S, Gascuel O. A simple, fast, and accurate algorithm to estimate large phylogenies by maximum likelihood. Syst Biol. 2003; 52(5):696–704. Epub 2003/10/08. https://doi.org/10.1080/10635150390235520 PMID: 14530136.

32. Junier T, Zdobnov EM. The Newick utilities: high-throughput phylogenetic tree processing in the UNIX shell. Bioinformatics. 2010; 26(13):1669–70. Epub 2010/05/18. https://doi.org/10.1093/bioinformatics/btq243 PMID: 20472542; PubMed Central PMCID: PMC2887050.

33. Gouy M, Guindon S, Gascuel O. SeaView version 4: A multiplatform graphical user interface for sequence alignment and phylogenetic tree building. Mol Biol Evol. 2010; 27(2):221–4. https://doi.org/10.1093/molbev/msp259 PMID: 19854763.

34. Wu WW, Phue JN, Lee CT, Lin C, Xu L, Wang R, et al. Robust Sub-nanomolar Library Preparation for High Throughput Next Generation Sequencing. BMC Genomics. 2018; 19(1):326. https://doi.org/10.1186/s12864-018-4677-y PMID: 29728062; PubMed Central PMCID: PMC5935984.

35. Santana-Quintero L, Dingerdissen H, Thierry-Mieg J, Mazumder R, Simonyan V. HIVE-hexagon: high-performance, parallelized sequence alignment for next-generation sequencing data analysis. PLoS One. 2014; 9(6):e99033. https://doi.org/10.1371/journal.pone.0099033 PMID: 24918764; PubMed Central PMCID: PMC4053384.

36. Simonyan V, Chumakov K, Dingerdissen H, Faison W, Goldweber S, Golikov A, et al. High-performance integrated virtual environment (HIVE): a robust infrastructure for next-generation sequence data analysis. Database (Oxford). 2016;2016. Epub 2016/03/19. https://doi.org/10.1093/database/baw022 PMID: 26989153; PubMed Central PMCID: PMC4795927.

37. Konduru K, Bradfute SB, Jacques J, Manangeeswaran M, Nakamura S, Morshed S, et al. Ebola virus glycoprotein Fc fusion protein confers protection against lethal challenge in vaccinated mice. Vaccine. 2011; 29(16):2968–77. https://doi.org/10.1016/j.vaccine.2011.01.113 PMID: 21329775; PubMed Central PMCID: PMC3070761.

38. Parren PW, Geisbert TW, Maruyama T, Jahrling PB, Burton DR. Pre- and postexposure prophylaxis of Ebola virus infection in an animal model by passive transfer of a neutralizing human antibody. J Virol. 2002; 76(12):6408–12. https://doi.org/10.1128/jvi.76.12.6408-6412.2002 PMID: 12021376; PubMed Central PMCID: PMC136210.

39. Mehedi M, Falzarano D, Seebach J, Hu X, Carpenter MS, Schnittler HJ, et al. A new Ebola virus nonstructural glycoprotein expressed through RNA editing. J Virol. 2011; 85(11):5406–14. Epub 2011/03/18. https://doi.org/10.1128/JVI.02190-10 PMID: 21411529; PubMed Central PMCID: PMC3094950.

40. Meyer M, Malherbe DC, Bukreyev A. Can Ebola Virus Vaccines Have Universal Immune Correlates of protection? Trends Microbiol. 2019; 27(1):8–16. https://doi.org/10.1016/j.tim.2018.08.008 PMID: 30201511; PubMed Central PMCID: PMC6309495.

41. Dietzel E, Schudt G, Krahling V, Matrosovich M, Becker S. Functional Characterization of Adaptive Mutations during the West African Ebola Virus Outbreak. J Virol. 2017; 91(2). https://doi.org/10.1128/JVI.01913-16 PMID: 27847361; PubMed Central PMCID: PMC5215343.

42. Raftery P, Condell O, Wasunna C, Kpaka J, Zwizwai R, Nuha M, et al. Establishing Ebola Virus Disease (EVD) diagnostics using GeneXpert technology at a mobile laboratory in Liberia: Impact on outbreak response, case management and laboratory systems strengthening. PLoS Negl Trop Dis. 2018; 12(1): e0006135. https://doi.org/10.1371/journal.pntd.0006135 PMID: 29304039; PubMed Central PMCID: PMC5755746.

43. Broadhurst MJ, Brooks TJ, Pollock NR. Diagnosis of Ebola Virus Disease: Past, Present, and Future. Clin Microbiol Rev. 2016; 29(4):773–93. https://doi.org/10.1128/CMR.00003-16 PMID: 27413095; PubMed Central PMCID: PMC5010747.

44. Audet J, Wong G, Wang H, Lu G, Gao GF, Kobinger G, et al. Molecular characterization of the monoclonal antibodies composing ZMAb: a protective cocktail against Ebola virus. Sci Rep. 2014; 4:6881. Epub 2014/11/07. https://doi.org/10.1038/srep06881 PMID: 25375093; PubMed Central PMCID: PMC5381473.

45. Davidson E, Bryan C, Fong RH, Barnes T, Pfaff JM, Mabila M, et al. Mechanism of Binding to Ebola Virus Glycoprotein by the ZMapp, ZMAb, and MB-003 Cocktail Antibodies. J Virol. 2015; 89(21):10982–92. https://doi.org/10.1128/JVI.01490-15 PMID: 26311869; PubMed Central PMCID: PMC4621129.

46. Konduru K, Shurtleff AC, Bavari S, Kaplan G. High degree of correlation between Ebola virus BSL-4 neutralization assays and pseudotyped VSV BSL-2 fluorescence reduction neutralization test. J Virol Methods. 2018; 254:1–7. https://doi.org/10.1016/j.jviromet.2018.01.003 PMID: 29355585; PubMed Central PMCID: PMC5826800.

47. Lee JE, Fusco ML, Hessell AJ, Oswald WB, Burton DR, Saphire EO. Structure of the Ebola virus glycoprotein bound to an antibody from a human survivor. Nature. 2008; 454(7201):177–82. Epub 2008/07/11. https://doi.org/10.1038/nature07082 PMID: 18615077; PubMed Central PMCID: PMC2700032.

48. Quinlan DS, Raman R, Tharakaraman K, Subramanian V, Del Hierro G, Sasisekharan R. An inter-residue network model to identify mutational-constrained regions on the Ebola coat glycoprotein. Sci Rep. 2017; 7:45886. https://doi.org/10.1038/srep45886 PMID: 28397835; PubMed Central PMCID: PMC5387726.

49. Miller CR, Johnson EL, Burke AZ, Martin KP, Miura TA, Wichman HA, et al. Initiating a watch list for Ebola virus antibody escape mutations. PeerJ. 2016; 4:e1674. Epub 2016/03/01. https://doi.org/10.7717/peerj.1674 PMID: 26925318; PubMed Central PMCID: PMC4768679.

50. Uyeki TM, Mehta AK, Davey RT Jr., Liddell AM, Wolf T, Vetter P, et al. Clinical Management of Ebola Virus Disease in the United States and Europe. N Engl J Med. 2016; 374(7):636–46. https://doi.org/10.1056/NEJMoa1504874 PMID: 26886522; PubMed Central PMCID: PMC4972324.

51. Fox EJ, Reid-Bayliss KS, Emond MJ, Loeb LA. Accuracy of Next Generation Sequencing Platforms. Next Gener Seq Appl. 2014;1. Epub 2015/02/24. https://doi.org/10.4172/jngsa.1000106 PMID: 25699289; PubMed Central PMCID: PMC4331009.
